# Amplification-free detection of SARS-CoV-2 using gold nanotriangles functionalized with oligonucleotides

**DOI:** 10.1007/s00604-022-05272-y

**Published:** 2022-04-01

**Authors:** Rafael del Caño, Tania García-Mendiola, Daniel García-Nieto, Raquel Álvaro, Mónica Luna, Hernán Alarcón Iniesta, Rocío Coloma, Ciro Rodríguez Diaz, Paula Milán-Rois, Milagros Castellanos, Melanie Abreu, Rafael Cantón, Juan Carlos Galán, Teresa Pineda, Félix Pariente, Rodolfo Miranda, Álvaro Somoza, Encarnación Lorenzo

**Affiliations:** 1 Departamento de Química Analítica, Universidad Autónoma de Madrid, 28049 Madrid, Spain; 2 Departamento de Química Física Y Termodinámica Aplicada e Instituto Universitario de Nanoquímica, Universidad de Córdoba, 14014 Córdoba, Spain; 3 Institute for Advanced Research in Chemical Sciences (IAdChem), Universidad Autónoma de Madrid, Ciudad Universitaria de Cantoblanco, 28049 Madrid, Spain; 4 Instituto de Micro Y Nanotecnología IMN-CNM, CSIC (CEI UAM+CSIC), Isaac Newton 8, Tres Cantos, 28760 Madrid, Spain; 5 IMDEA-Nanociencia, Ciudad Universitaria de Cantoblanco, 28049 Madrid, Spain; 6 Servicio de Microbiología, Hospital Universitario Ramón Y Cajal and Instituto Ramón Y Cajal de Investigación Sanitaria (IRYCIS), 28034 Madrid, Spain; 7 Red Española de Investigación en Patología Infecciosa (REIPI), Instituto de Salud Carlos III, Madrid, Spain; 8 Centro de Investigación Biomédica en Red (CIBER) en Epidemiología Y Salud Pública, Instituto de Salud Carlos III, Madrid, Spain

**Keywords:** AuNTs, Biosensor, SARS-CoV-2, Amplification-free detection

## Abstract

Gold nanotriangles (AuNTs) functionalized with dithiolated oligonucleotides have been employed to develop an amplification-free electrochemical biosensor for SARS-CoV-2 in patient samples. Gold nanotriangles, prepared through a seed-mediated growth method and exhaustively characterized by different techniques, serve as an improved electrochemical platform and for DNA probe immobilization. Azure A is used as an electrochemical indicator of the hybridization event. The biosensor detects either single stranded DNA or RNA sequences of SARS-CoV-2 of different lengths, with a low detection limit of 22.2 fM. In addition, it allows to detect point mutations in SARS-CoV-2 genome with the aim to detect more infective SARS-CoV-2 variants such as Alpha, Beta, Gamma, Delta, and Omicron. Results obtained with the biosensor in nasopharyngeal swab samples from COVID-19 patients show the possibility to clearly discriminate between non-infected and infected patient samples as well as patient samples with different viral load. Furthermore, the results correlate well with those obtained by the gold standard technique RT-qPCR, with the advantage of avoiding the amplification process and the need of sophisticated equipment.

**Graphical abstract:**

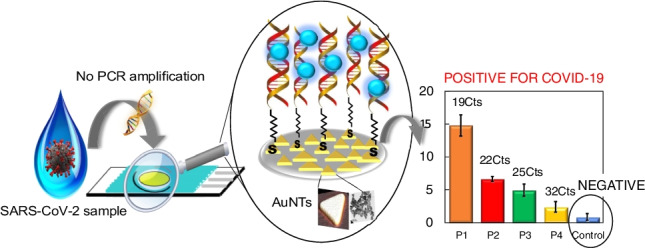

**Supplementary Information:**

The online version contains supplementary material available at 10.1007/s00604-022-05272-y.

## Introduction

The great impact caused by the SARS-CoV-2 coronavirus, and its variants (Alpha, Beta, Gamma, Delta, and Omicron), are being enormous not only in the global health but also in the economy. The efforts to contain the virus are ongoing, and currently, it is known that a critical point to achieve it relies on the ability to diagnose the disease rapidly and accurately. At present, COVID-19 is being primarily diagnosed by quantitative reverse-transcription polymerase chain reaction (RT-qPCR) or rapid antigen point-of-care tests test which is a lateral flow immunoassay that detects the presence of SARS-CoV-2 proteins in patient samples.

The RT-qPCR is the golden standard diagnostic method for the detection and screening of the SARS-CoV-2. However, this tool takes large time of analysis, advanced instrumental techniques, and qualified personnel. Additionally, while point-of-care tests are rapid and require minimal equipment, their efficacy when the viral load is low is limited. Therefore, the development of alternative systems for virus detection and its variants is increasing to address these problems. In this sense, reliable biosensor systems for the detection of this virus could be an interesting alternative to focus on since they present simplicity, low cost, high sensitivity, and specificity. In fact, they are in urgent demand for the control of the SARS-CoV-2 pandemic.

Designs of a biosensor platform for the detection of SARS-CoV-2 involve the following aspects: (1) the target of recognition, such as viral RNA, a viral protein, or human immunoglobulins; (2) the recognition method, such as through nucleic acid probes, aptamers, antibodies, enzymatic reactions; (3) the transduction and signal amplification system, such as electrochemical, optical, electrical, mechanical, among others. Aspects (2) and (3) must be closely related to each other to enhance the performance of the final device.

Biosensors based on antibody-antigen binding, although very attractive, require high quantities of purified antibodies and virus titers in samples [1–4]. In contrast, nucleic acid biosensors are emerging as a new alternative since they can achieve the sensitivity of RT-qPCR and the simplicity and rapidity of antigen point-of-care tests. These DNA biosensors are based on complementary DNA or RNA molecules that can hybridize selected sequences from the target analyte. The standard target genes of the SARS-CoV-2 include the specific viral gene regions coding for the RNA-dependent RNA polymerase (RdRp), nucleocapsid protein (N), spike protein (S), and the open reading frame 1 ab (ORF1ab) sequences. All of them can be employed for the development of DNA biosensors for SARS-CoV-2. Among the different transduction techniques, electrochemical devices, due to their rapid detection efficacy and ease of use for point of care applications, are starting to appear in the literature for the detection of SARS-CoV-2 [5–11]. These biosensors present analytical features such as time of response between 3 and 1 h and detection limits from 10 nM to 3aM. However, in the case of the articles with lower detection limits, the strategies used are quite complicated and require many steps increasing analysis time. Hence, one of the most challenges developing new device for SARS-CoV-2 detection is the possibility of detecting this virus when the viral load is low as RT-qPCR does, using rapid, simple strategies and avoiding amplification process. In this sense, nanotechnology and the use nanomaterials can improve not only in the performance of electrochemical biosensors concerning sensitivity, selectivity, small size, and simplicity of the final device but also in several aspects of the fight against the virus [12]. On the base of these premises, we decided to utilize exclusive features of nanomaterials in developing a disposable electrochemical DNA biosensor based on gold nanotriangles for the sensitive and specific detection of SARS-CoV-2 avoiding amplification procedures.

Gold nanostructures such as gold nanoparticles (AuNPs) are ideal platforms for electrochemical biosensors due to their excellent conductivity, high surface area, proper biocompatibility, and eminent catalytic activity [13, 14]. Despite the considerable interest in gold nanostructures, the use of different shapes of gold nanomaterial as gold nanotriangles has not been widely exploited yet. Only few reports have studied the influence of shape on the biosensor development. For instance, Abedi et al. have reported the use of gold nanocubes (AuNCs) in the development of an electrochemical DNA biosensor. Compared to AuNPs, gold nanocubes (AuNC) tend to increase biosensor stability. In addition, the edges and vertices of AuNC provide higher surface energy, so there are more reaction sites available, resulting in more reactive facets [15, 16]. Furthermore, Bollalla et al. [17] have also observed that the shape of the gold nanomaterials has a crucial effect on the biocatalytic current related to the oxidation of fructose. In particular, they have observed that gold nanotriangles (AuNTs) have a higher effect compared with the spherical one. This phenomenon is ascribed to the more effective enzyme-nanomaterial interaction provided by the edge of the triangle, and the higher number of enzyme molecules that can be allocated on the electrode surface. In the same sense, Liebig et al. have observed that the sharpness of the edges and tips of AuNTs is of special relevance for improving the enhancement factor, and therefore, the application in catalysis and sensing [18]. Despite these findings would be a subject to study of great interest in the development of new improved electrochemical biosensor, as far as we know, the advantages of use gold nanotriangles (AuNTs) as electrode modifiers in the development of electrochemical DNA biosensors have not studied yet. Therefore, we decide to prepare an electrochemical biosensor for SARS-CoV-2 detection based on AuNTs as electrode modifiers.

In this biosensor, the probe-DNA hybridization is conducted to determine a specific DNA or RNA sequence of the target genes of the virus, by immobilizing the corresponding thiol modified oligonucleotide on the gold triangle's surface. Hybridization event is detected using Azure A as electrochemical indicator, which presents the advantage of low redox potential (avoiding interferences) and less unspecific adsorption on the electrode surface than other classic dyes as methylene blue or neutral red. The method provided a trustworthy approach to directly realize the detection of DNA or RNA specific region sequences from RdRp and spike protein (Spike) region genes of SARS-CoV-2. It has been successfully applied to detect SARS-CoV-2 in nasopharyngeal swab samples from COVID-19 patients without any amplification process.

## Material and methods

### Chemicals

Sodium thiosulphate (Na_2_S_2_O_3_), tetrachloroauric acid (HAuCl_4_), sodium chloride, sodium phosphate, Azure A, and all other chemicals used in this work were obtained from Merck. (www.merckgroup.com/es-es). Water was purified with a Millipore Milli-Q-System (18.2 MΩ cm).

#### DNA/RNA samples

Short DNA and RNA sequences were synthesized in a H-6/H-8 DNA/RNA Synthesizer (K&A) and purified using LCG Biosearch Micropure purification columns, as described in the supporting information. Long DNA and RNA sequences were obtained by enzymatic expression as detailed in the supporting information. These sequences are summarized in Table 1SI.

RNA samples from patients were extracted using the QIAamp Viral RNA kit from Qiagen. The DNA/RNA SARS-CoV-2 samples used in this work are listed in Table 1SI and comprise capture probes, analyte sequences, and non-complementary (control) sequences. The capture probes are single-stranded sequences complementary to the analyte, a specific DNA or RNA sequence from RNA dependent RNA polymerase (RdRP) gene and from spike protein (Spike) region gene. The target analytes comprise specific different lengths (25, 72, and 100 nucleotides) RNA and DNA sequences of both genes. The non-complementary (control) sequences correspond to the sarbecovirus envelope gene (denoted in the text as CoV-2-E-NC-DNA-25), a region encoding for the green fluorescent protein (GFP) (denoted in the text as GFP- NC-DNA72) and a region of the antisense RNA from RdRP gene (CoV-2-R-NC-RNA126) with the same sequence polarity as for the capture probe.

#### COVID-19 patient samples

Four nasopharyngeal swab samples were used. Three of them from SARS-CoV-2 infected patients (P1, P2, P3 and P4), and the other from a non-infected patient used as a control. The viral charge of SARS-CoV-2 in the samples was determined by RT-qPCR. (Ct values of 19, 22, 25, and 32 for P1, P2, P3, and P4, respectively).

##### Ethics approval

The samples were obtained with the consent all participants and approved by “Comité de Ética de la Investigación con Medicamentos del Hospital Universitario Ramón y Cajal”. Reference: 127–21.

### Apparatus

Electrochemical experiments were performed using an Autolab (PGSTAT 30) potentiostat attached to a PC with proper software (GPES and FRA) for the total control of the experiments and data acquisition. A screen-printed electrode connector (DropSens) was used as interface. Screen-printed Carbon electrodes (CSPEs) were supplied by DropSens (Spain). CSPEs integrate a carbon working, silver pseudo-reference, and carbon counter electrodes.

All the apparatus employed to characterize the gold nanotriangles (AuNTs) synthetized and the nanostructured electrode surface are described in the Supporting information (SI).

### Procedures

#### Synthesis of gold nanotriangles

The synthesis of gold nanotriangles (AuNTs) was carried out according to a seed-mediated growth method [19] with some modifications of the reaction conditions to improve the yield, simplify the procedure, and to obtain isolated gold nanotriangles. This method is based on the reduction of HAuCl_4_ by Na_2_S_2_O_3_ in an aqueous medium. Briefly, the gold nanotriangles were prepared as follows. In a 100-mL Erlenmeyer flask, 30 mL of 0.5 mM Na_2_S_2_O_3_ was added to 25 ml of 2 mM HAuCl_4_, with vigorous stirring on a magnetic stirring hot plate. After 9 min, 12.5 mL of 0.5 mM Na_2_S_2_O_3_ was added to the solution all at once, with vigorous stirring. The yellow solution turned clear, brown, and then deep red within a few minutes. During these previous 9 min, the seeds are formed. After the second addition of thiosulfate, the growth of seeds and formation of the nanotriangles occur and stirring is maintained for 45 min. A mixture of spherical and triangular gold nanoparticles is obtained. To separate the triangular gold nanoparticles from the spherical seeds, a separation procedure was carried out using a surfactant-assisted depletion methodology in the presence of 0.167 M cetyltrimethylammonium bromide (CTAB) at 28 °C for 24 h. After this time, the AuNTs precipitate, while the supernatant maintains the reddish colour typical of spherical nanoparticle dispersions. Finally, the precipitate was redispersed in water, obtaining a dark green solution corresponding to the isolated AuNTs.

#### SARS-CoV-2 DNA/RNA sequence preparation

Sequence preparation is described in detail in Supporting Information.

Prior to use, stock solutions of thiol-modified probes (Probe-R-DNA-SH) were treated with DTT and then purified by elution through a NAP-10 column of Sephadex G-25. Afterwards, the stock solutions of the thiol probes were prepared at a final concentration of 10.0 µM in 10 mM phosphate buffer (PB) pH 7.0. In the case of dithiolane probes (Probe-R-DNA-dithio and Probe-S-dithio), there is no need for deprotection by DTT or further purification, and the stock solutions were also prepared at a final concentration of 10.0 µM in 10 mM phosphate buffer (PB) pH 7.0. The stock solutions of the analyte sequences were prepared in 10 mM phosphate buffer (pH 7.0) with 0.4 M NaCl. Aliquots of a few microliters of all stock solutions were stored at − 20 °C.

#### SARS-CoV-2 biosensor development

##### Electrode modification

CSPE were nanostructured with the as synthesized AuNTs (AuNT/CSPE) by spraying them onto the electrode surface with an airbrush for 60 s. During the process, the electrode is heated at 90 °C on a hot plate, which allows the fast evaporation of the solvent. Afterwards, 10 µL of 10.0 µM of the corresponding capture probe (Probe-R-DNA-thiol, Probe-R-DNA-dithio and Probe-S-DNA-dithio) were deposited on the AuNT/CSPE by drop-casting and was kept at room temperature for 24 h. Finally, it was soaked in Milli-Q water for 30 min. The resulting modified electrodes are named as Probe/AuNTs/CSPE.

##### Hybridization and electrochemical detection

The modified electrode was hybridized in 10 mM phosphate buffer (pH 7.0) supplemented with 0.4 M NaCl for 1 h at 40 °C with 10 µL of the analyte solution, which may contain a complementary sequence corresponding to SARS-CoV-2 (CoV-2-R-DNA25, CoV-2-R-DNA74, CoV-2-R-RNA100, CoV-2-S-DNA72, CoV-2-S-DNA72-SNP) or a non-complementary (CoV-2-E-NC-DNA25, GFP-NC-DNA72, CoV-2-R-NC-RNA126) sequence used as control. Electrochemical detection was carried out using Azure A (AA) as the electrochemical indicator. Twenty microliters of 10 mM AA chloride were dropped on to the surface of the modified and hybridized electrode for 30 min. Finally, the electrodes were rinsed with sterile water, placed in 0.1 M PB pH 7.0, and differential pulse voltammograms (DPVs) with a scan rate of 10 mVs^−1^ were immediately recorded. The biosensor response was the peak current measured at − 0.23 V (*vs* Ag ref. electrode) corresponding to the oxidation of the accumulated AA.

#### Amplification-free detection of SARS-CoV-2 from COVID-19 patients

Patient samples were obtained from Hospital Ramón y Cajal and were inactivated with guanidine isothiocyanate. RNA was extracted using QIAamp Viral RNA kit from Qiagen (Cat. No. 52906) according to manufacturer instructions. All the processes were carried out in BSL-2 hood and following all the recommendations and procedures for RNA preservation to avoid degradation of the samples or cross-contamination. Extraction columns were loaded with 300 µL patient samples and finally eluted in 30 µL RNase free water. The obtained RNA sample patients were aliquoted and stored at − 20 °C.

Five microliters of the sample were deposited on the electrode modified with the capture probe (Probe-R-DNA-dithio/AuNTs/CSPE) and were allowed to hybridize for 1 h at 40 °C. Finally, AA was accumulated on the hybridized DNA layer as we described above and DPVs were recorded.

#### Statistical analysis

The results are presented as the mean ± standard deviation (SD) (*n* = 3). The statistical analysis was carried out using the R software (R Development Core Team, Vienna, Austria) [20] When the means of only two groups were compared, a Student’s *T* test for independent samples, with a confidence interval of 95%, has been used. When the means of more than two groups were compared, we used the one-way analysis of variance (ANOVA) (Tukey’s test, pairwise comparison of multiple means).

## Results and discussion

### The working principle

**Scheme 1.**
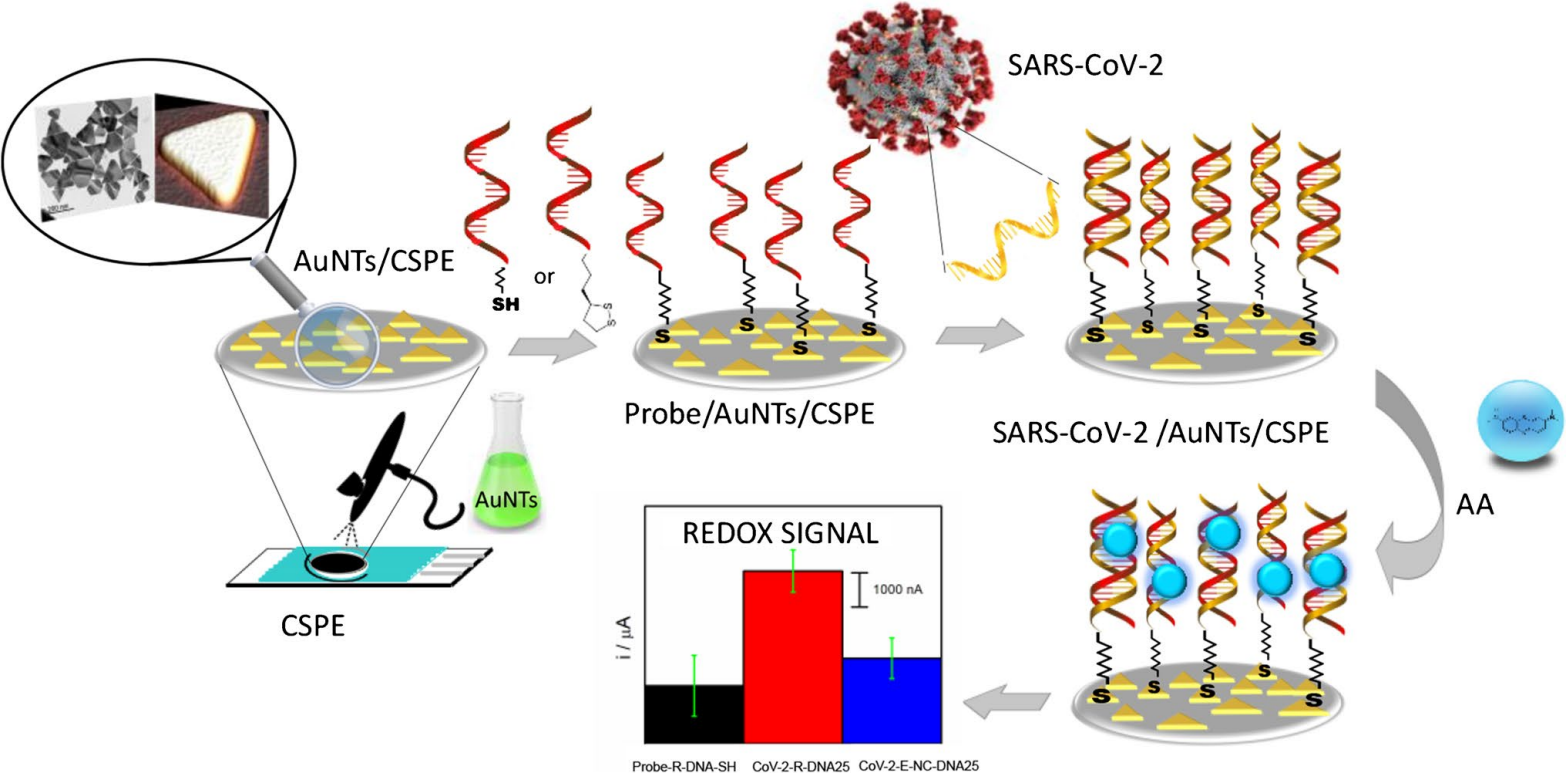
Design of the biosensor development

The principle of the electrochemical biosensor proposed is depicted in Scheme 1. We have engineered a simple and efficient electrochemical platform based on the deposition of gold nanotriangles (AuNTs) on carbon screen-printed electrodes (CSPEs) and further modification of these nanostructures with the capture probe, as base to develop a sensitive and selective strategy to detect different SARS-CoV-2 sequences to assess its broad applicability. As can be seen in Scheme 1, at a first step CSPE is modified with AuNTs. Then, the probe-target hybridization is conducted to detect specific DNA or RNA sequences from the RdRp and spike genes of SARS-CoV-2, by immobilizing the corresponding thiolated capture probe on the AuNTs surface. Finally, hybridization is detected using Azure A as redox indicator.

### Synthesis and characterization of AuNTs

**Fig. 1:**
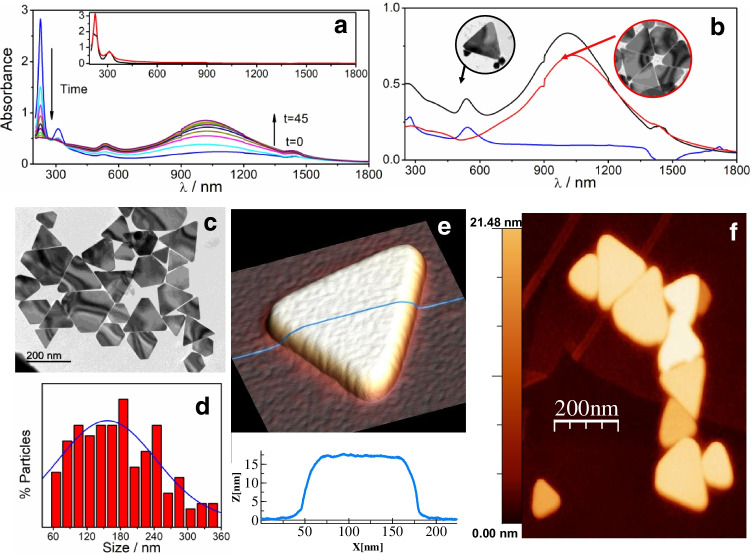
Changes in the UV–visible-NIR spectra for **a** the different stages of the seed-mediated procedure for AuNTs preparation **b** UV–visible-NIR spectra of the final reaction mixture by depletion: (black line) mixture, (red line) redispersed precipitate, (blue line) supernatant. The TEM images in the insets are for the mixture and the AuNTs after the separation. **c** TEM images of the AuNTs. **d** Histogram obtained by measuring different TEM images. **e** 3D AFM image of a single AuNT with a topographic profile taken along the blue line and **f** 2D AFM image of a group of AuNTs

Gold nanotriangles (AuNTs) were obtained according to a seed-mediated growth method with some modifications of the reaction conditions to improve the yield, the procedure and selection of gold nanotriangles. This method is based on the HAuCl_4_ and Na_2_S_2_O_3_ as precursors, as explained in detail in the Experimental section (Scheme 1SI). The course of the reaction was followed by UV–visible-NIR spectroscopy (Fig. 1a). The addition of Na_2_SO_3_ to the HAuCl_3_ solution produces the fast decrease of the characteristics HAuCl_3_ bands at 225 and 310 nm concomitant with the appearance of two bands at 539 and 1015 nm associated with the formation of spherical and triangular nanoparticles, respectively. The bands increased with time up to 45 min when the HAuCl_3_ has completely reacted. To separate the different nanostructures formed, the obtained mixture is submitted to a depletion procedure that uses CTAB and induces the precipitation of the AuNTs while keeps the spherical nanoparticles dispersed. The spectra in Fig. 1b evidence the success of the separation.

The obtained AuNTs were characterized by different techniques as transmission electron microscopy (TEM), dynamic light scattering (DLS), zeta potential, atomic force microscopy (AFM), and scanning electron microscopy (SEM) to elucidate their morphology, size, and properties. The morphology and dimensions of the AuNTs were determined by TEM. As shown in Fig. 1c, isolated AuNTs present mainly a triangular morphology with diameters distributed in a range from 50 to 360 nm and an average lateral length of 156 nm ± 0.5 nm, as can be seen in the histogram of Fig. 1d. The AFM study of AuNTs deposited on Highly Oriented Pyrolytic Graphite (HOPG) provides height information in addition to confirming their triangular morphology (Fig. 1d and e). The profile in Fig. 1e (blue line) shows an AuNT average height of 17 nm. AuNTs with average heights between 14 and 21 nm can be found in Fig. 1f. The morphologic AuNTs characteristics found by SEM (see Fig. 1a, b, c, and d of SI) are in good agreement with those observed by TEM and AFM.

Zeta potential of the AuNTs of around 35 mV is measured in a wide pH range. This high positive value is due to the presence of CTAB protecting the surface that is also responsible for its high stability of the aqueous dispersion against aggregation, as demonstrated by DLS (Fig. 1e and f of SI).

Therefore, based on the results described above, we can confirm the successful synthesis and isolation of the AuNTs.

### Development and characterization of the biosensor

As can be seen in Scheme 1, the first step in the biosensor development was the nanostructuration of the electrode surface using the synthesized AuNTs. Two different strategies were carried out to achieve this process, drop-casting or spraying the nanomaterial. The latest strategy allows nanomaterial deposition on the electrode surface by using an airbrush and applying heat to the CSPE at the same time (see experimental section), giving a more homogeneous and reproducible surface. After this process, the corresponding capture probe, a single-stranded DNA sequence complementary to the target analyte, modified at 3′-end with a thiol moiety (Probe-thiol) or dithiolane moiety (Probe-dithio), was immobilized on the nanostructured electrode surface (AuNTs/CSPE). The organic molecules with -SH group tend to react covalently with gold, resulting in the molecular functionalization of the AuNTs/CSPE. Characterization of the biosensing platform was performed by scanning electron microscopy (SEM–EDX) and Cyclic Voltammetry (CV).

**Fig. 2:**
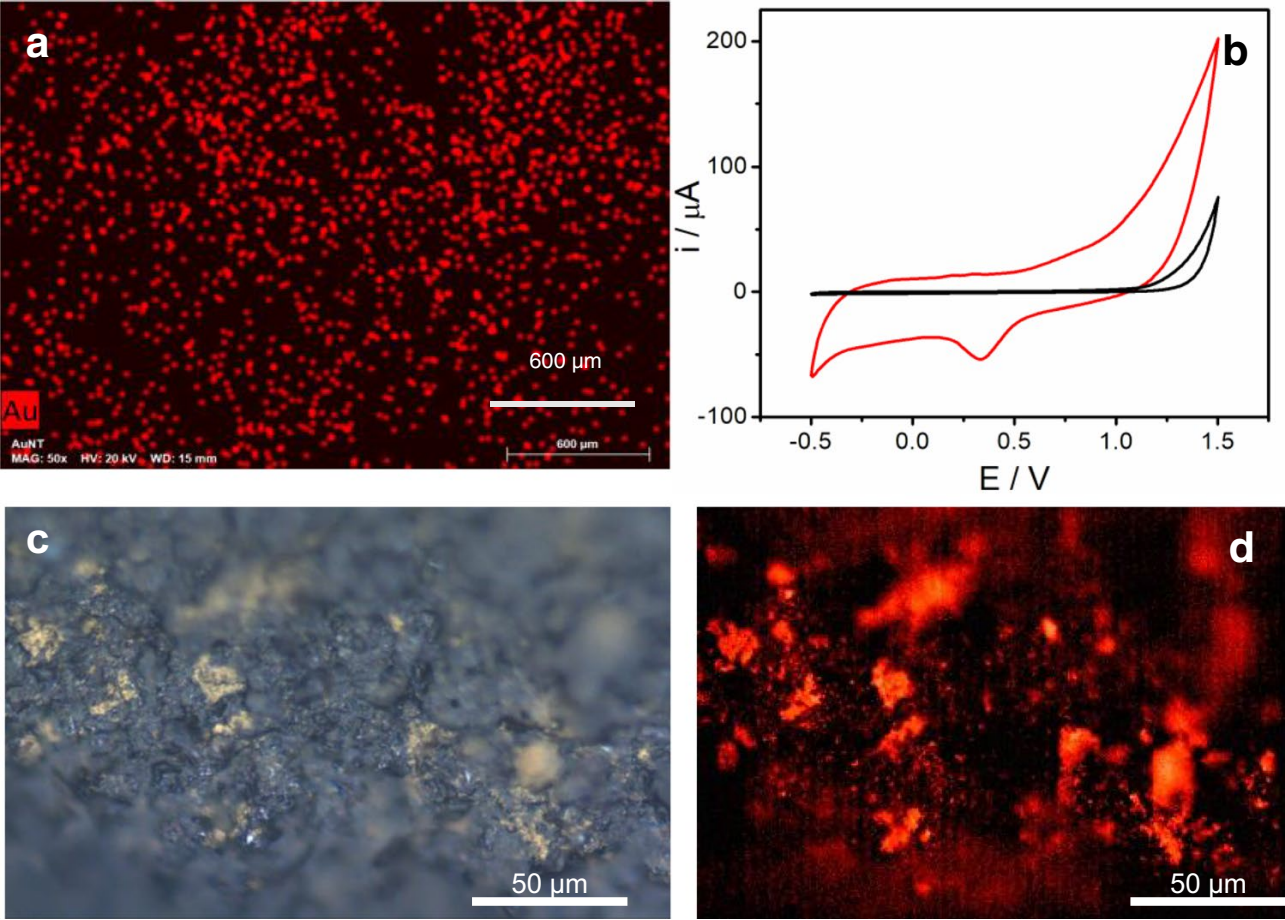
**a** SEM–EDX image of a magnification of AuNTs/CSPE. Scale bar corresponds to 600 µm. **b** Cyclic voltammograms from − 0.5 to 1.5 V (*vs* Ag ref. electrode) of a CSPE (black line) and an AuNTs/CSPE (red line) in 0.1 M H_2_SO_4_. Scan rate: 100 mVs^−1^. **c** Optical microscope white light and **d** fluorescence images of TAMRA-Probe-SH/AuNTs /CSPE. Fluorescence signal is only detected in the yellowish areas (left) where Probe-SH/ AuNTs are present

The SEM image of the bare electrode (see Fig. 2 of SI) shows a rough surface that, according to the EDX spectrum, is mainly composed of carbon. After AuNTs modification (Fig. 2a), a homogeneous distribution of the AuNTs onto the CSPE surface can be observed. The EDX spectrum (Fig. 2c of SI) confirms the presence of Au. Cyclic voltammograms after the AuNTs modification process (Fig. 2b) show the characteristic peaks of the gold, confirming again CSPE modification with AuNTs. From the reduction peak of gold oxide, the electroactive surface area was estimated to be 0.013 cm^2^.

Immobilization of the thiol or dithiolane capture probe on the AuNTs modified electrodes (Probe-thiol/AuNTs/CSPE or Probe-dithio/AuNTs/CSPE, respectively) was also assessed by fluorescence microscopy and CV.

**Fig. 3:**
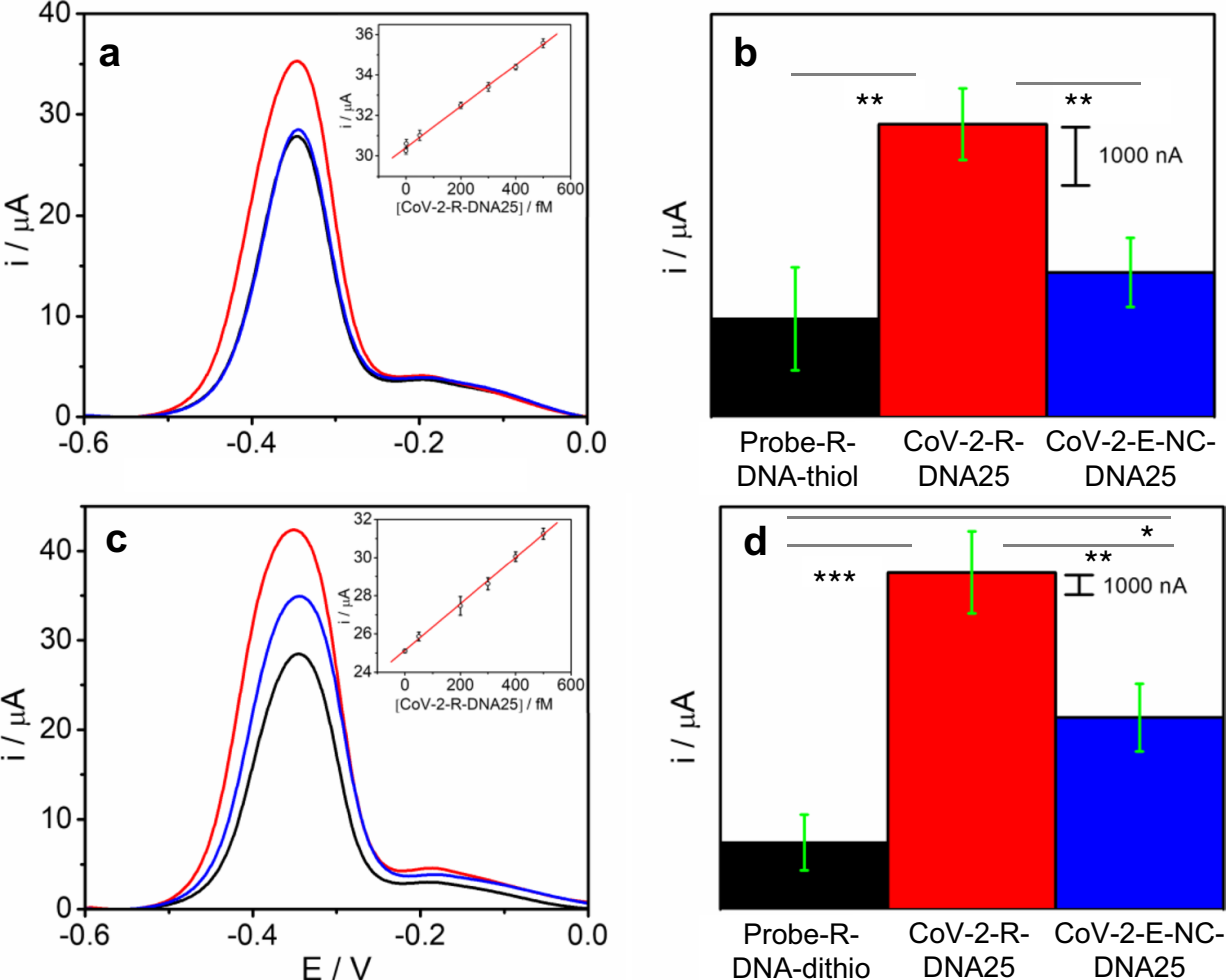
DPVs (**a**, **c**), bar diagrams (**b** and **d**), and calibration plots (inset of **a** and **c**) of the peak current of AA accumulated (measured at − 0.23 V (vs Ag ref. electrode)) on a Probe-R-DNA- thiol/AuNT/CSPE (**a**, **b**) or Probe-R-DNA-dithio/AuNT/CSPE (**c**, **d)** before (black line) and after hybridization with 500 fM: complementary CoV-2-R-DNA25 (red line) and a non-complementary CoV-2-E-NC-DNA25 (blue line) sequences in PB 0.1 M pH 7.0. Scan rate: 10 mV s^−1^. Data are presented as mean ± SD (*n* = 3) in terms of intensity. Statistical analysis was performed using one-way ANOVA with Tukey’s test (pairwise comparison). Significant codes: 0 “***” 0.001 “**” 0.01 “*” 0.05 “.” 0.1 “” 1 “.”

In the case of fluorescence microscopy studies, a probe modified with a fluorophore (TAMRA or tetramethylrhodamine) was used (Probe-R-TAMRA-SH) to follow the AuNTs/CSPE probe immobilization. Yellowish patches surrounded by grey colour can be observed on the white light optical microscope image (Fig. 2C). They correspond to the areas where Probe-R-TAMRA-SH have been deposited on AuNTs/ CSPE. In the fluorescence image of the same area (Fig. 2d), the signal can be detected only where Probe-R-TAMRA-SH/AuNTs/CSPE is present. Bare AuNTs on CSPE do not show any fluorescence contrast (Fig. 3SI). This result confirms that thiolated DNA probe is specifically bound to AuNTs. Besides acting as supporting to DNA probe, AuNTs increase the specific electrode surface and improves the electron transfer, which results in a higher current response. In addition, it has been reported that the geometry of the nanostructure plays a key role in improving the electron transfer in bioelectrocatalytic reactions [17]. We expect that AuNTs have a self-packing pattern onto the electrode surface different to AuNPs, resulting in a different real surface area [21].

We mentioned that we employed Azure A (AA) as redox indicator of the hybridization event. Hence, we studied the CV response of AA at a CSPE, an AuNTs /CSPE, and a probe-thiol/ AuNTs /CSPE, (see Fig. 4 of SI). In all cases, it can be observed the characteristic pair of redox peaks, ascribed to the oxidation/reduction of the AA [22] at a formal potential (E°′) value of − 0.33 (vs Ag ref. electrode) with a peak potential separation (ΔEp) of 59.5 mV. As one would expect, the presence of AuNTs causes an increase in the peak current, concomitant with a slight shift to more negative formal potentials and an increase in the ΔEp of 44 mV. When the electrode is modified with the thiolated probe (Probe-R-DNA- thiol/AuNTs /CSPE), the peak of AA shifts 20 mV to positive values, which is indicative of an interaction of the AA and the DNA immobilized on the electrode surface [23] and compatible with the immobilization of the capture probe on the AuNTs /CSPE.

### SARS-CoV-2 detection

As we described above, we have applied the biosensor to the detection of different sequences from RdRp gene of SARS-CoV-2. In a first attempt, 25 nts DNA sequences from RdRP gene of SARS-CoV-2 were used as a prototype system. As a capture probe, we use a complementary sequence modified on 3′ extreme with a thiol (see Table 1) to achieve the probe immobilization on the AuNT surface. After immobilization of the DNA probe, the hybridization with the target analyte was carried out on the biosensor surface. Azure A was accumulated on the dsDNA formed after hybridization and employed as an electrochemical indicator of the hybridization event [22].

**Table 1:** Comparative electrochemical biosensors for SARS-CoV-2 detection

*Target analyte*	*Probe*	*Principle*	*Analysis Time*	*Mediator*	*L.O.D*	*Reference*
*26-nt-long ORF1ab fragment of SARS-CoV-2 RNA*	Hairpin 1 and 2	Catalytic hairpin assembly	DPV (3 h)	Ru(NH_3_)_6_^3+^	26 fM	[10]
*25-nt-long ORF1ab fragment of SARS-CoV-2 RNA*	Thiolated DNA	MoS_2_ as platform for thiolated DNA probe immobilization and thionine-carbon nanodots as electrochemical indicator	DPV (2 h)	Thi-CNDs	1.01 pM	[11]
*SARS-CoV-2 RNA-dependent RNA polymerase (RdRp) gene*	Thiol-modified primers	Electrochemical biosensor combined with recombinase polymerase amplification (RPA)	DPV (-)	Potassium ferricyanide (K_3_[Fe(CN)_6_])	0.972 fg/μL	[6]
*SARS-CoV-2 RNA-dependent RNA polymerase (RdRp) gene*	Functionalized amine groups with probe DNA	Interdigitated platinum/titanium electrodes	Impedance (2 h)	-	10 nM	[7]
*COVID-19 N-gene*	Especific biotin-labeled probes	Electropolymerized polyaniline (PANI) nanowires and newly designed peptides	DPV (1 h)	PANI polymer	3.5 fM	[8]
*Open reading frame (ORF1ab) from SARS-CoV-2*	Thiolated DNA	Supersandwich-type recognition strategy with calixarene	DPV (3 h)	Toluidine blue	3 aM	[9]
*SARS-CoV-2 RdRp and Spike genes*	Dithiolated DNA and RNA	Gold nanotriangles (AuNTS)/electrochemical indicator	DPV (1.5 h)	Azure A	22.2 fM	This work

The experimental parameters, including electrolyte, ionic strength, temperature, and time of hybridization, were optimized. From these studies, it can be concluded best results were obtained when hybridization was carried out for 1 h at 40 °C in 10 mM phosphate pH 7.0 with 0.4 M NaCl.

To choose the best strategy to incorporate the redox indicator in the dsDNA layer, AA accumulation was carried out just by adsorption or electrochemically driven by cycling the potential from − 0.8 to 0.3 V (*vs* Ag ref. electrode). Both strategies allow AA accumulation (see Fig. 5SI). However, adsorption not only gives a larger accumulation; it is also a simpler procedure. Therefore, accumulation of the redox indicator was carried out by adsorption, dropping it to the electrode surface, and let to react for 30 min.

Figure 3a shows the signal of the AA accumulated on the CoV-2-R-DNA25/Probe-R-DNA- thiol/AuNTs/CSPE obtained before (a) and after the hybridization (b) with the complementary sequence. As can be observed, after hybridization the redox signal is much higher (38µA) than that obtained for the probe (28µA).

The analytical parameters of the proposed DNA biosensor were studied under the experimental conditions previously selected as optimal and measuring the biosensor signal at − 0.23 V (vs Ag ref. electrode). Figure 3b and 6 of SI shows the biosensor’s response to increasing amounts of SARS-CoV-2 sequence. It can be observed that the response increases as the concentration of SARS-CoV-2 sequence does. This increase is linear (*r*^2^ = 0.993) in the concentration range studied, with a slope of 0.0102 (µAfM^−1^) and an intercept of 30.4 ± 0.02 (µA). The limit of detection (LOD) and quantification (LOQ) were estimated using the 3 Sb m^−1^ and 10 Sb m^−1^ criteria, respectively, where *m* is the slope of the calibration curve and *Sb* is the standard deviation of the background signal (probe-SH/ AuNTs /CSPE). Values of 36.3 and 158 fM were calculated for the limit of detection (LOD) and quantification (LOQ), respectively.

Immobilization of the capture probe plays a crucial role in the biosensor design since the hybridization event and, therefore the sensitivity and operational life of the device depend on this step. Hence, to improve the probe immobilization we have developed capture probes modified with a dithiolane moiety at the 3′ end of the sequence (see Table 1) since we assumed that the dithiolane moiety facilitates the conjugation with gold. The electrochemical response of the biosensor before and after the hybridization with the complementary sequence, at the same optimal conditions described above, is shown in Fig. 3 (c and d). Remarkably, when the dithiolane probe was used (using the same immobilization conditions that in the case of the thiol probe), the difference in the electrochemical signal before and after the hybridization step was 40% higher than in the case of the previous standard thiolated probe. This improvement in the biosensor response seems to indicate that the dithiolane probe provides a DNA sensing layer more effective than the thiolane probe, probably due to the immobilization of the dithiolated probes results in greater stability and more appropriate spatial conformation to hybridization than the thiolated ones. The analytical parameters of the DNA biosensor prepared using a dithiolane sequence were also studied (Fig. 3c and 6 of SI). In this case, the linear equation obtained was *Y* = 0.0121 [SARS-CoV-2] + 25.14 (*R* = 0.9996). The method has a sensitivity of 0.0121 µAfM^−1^, obtained from the slope of the plot. The LOD and LOQ were found to be 22.2 and 75.7 fM, respectively. Moreover, the use of the dithiolane moiety in the probes does not require the previous deprotection by DTT, purification and concentration before use, and the time required for the modification of the electrodes was also shorter. Therefore, due to these significant improvements compared with the thiolated probes, the dithiolane probe was chosen for biosensor development and in the following work.

The selectivity of the biosensor was assessed using a non-complementary sequence corresponding to the envelope gene (denoted in the text as CoV-2-E-NC-DNA-25) as target. As shown in Fig. 3c and d, when the hybridization occurs with the non-complementary sequence, a response very similar to that of the probe is observed. These results suggest that no hybridization process is taking place; the slight increase observed is probably due to nonspecific adsorption. Statistical analysis also confirms that significant differences are observed between the probe and the complementary sequence as well as between the non-complementary and the complementary sequence. The reproducibility of the method was estimated from the response of three devices, prepared using the same protocol, to 500 fM analyte sequence. The relative standard deviation (RSD) was calculated to be 0.8%. The repeatability of the biosensor was also evaluated by measuring 5 times the biosensor response. In this case, the RSD was found to be 0.25%.

The biosensor response is almost stable over a period of one month. The selectivity of the biosensor was also evaluated. For this purpose, the response to samples containing the SARS-CoV-2 sequence (50.0 pM) in the absence or in the presence of other virus sequences as SARS-CoV-1and Influenza A (H7N9) (final concentration of 50.0 pM) was recorded. As can be seen (Fig. 7 of SI) the presence of other potentially interfering virus sequences does not alter the biosensor response to SARS-CoV-2.

To prove that the nanomaterial geometry plays a key role on the biosensor performance, we also developed the biosensor using the same methodology but spherical gold nanoparticles (AuNPs) instead of AuNTs as gold nanostructures. In this case, based on the statistical analysis performed using Student’s *T* test, there are non-significant (n.s) differences in the peak current obtained for complementary (CoV-2-R-DNA25) or non-complementary (CoV-2-E-NC-DNA-25) sequences when the concentration is 500 fM (see Fig. 8SI). However, significant (sig) statistical differences were observed at higher concentrations (at least 100 pM). The analytical parameters of the biosensor were also studied. The response increases linearly (*y* = 0.025 [SARS-CoV-2] + 22.47 (*R* = 0.997)) as the concentration of SARS-CoV-2 sequence does (see Fig. 9SI). The sensitivity, obtained from the slope of the plot, was found to be of 0.025 µAfM^−1^. A LOD of 360 fM was calculated, being much higher than in the case of AuNT-nanostructured biosensor. These results confirm that the shape of the nanostructure plays a crucial role and the proposed methodology, based on the use of AuNTs modified electrodes, works on recognizing specific DNA sequences better than AuNPs.

#### Detection of longer SARS-CoV-2 sequences

**Fig. 4:**
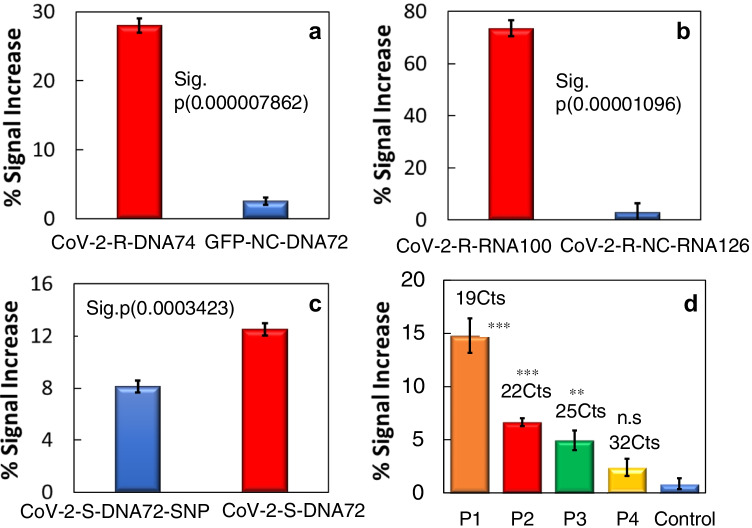
Bar diagrams of the % signal increase after the hybridization with 50 fM: **a** 74 nts CoV-2-R-DNA74 complementary sequence or GFP-NC-DNA72 non-complementary sequence; **b** CoV-2-R-RNA100 sequence or CoV-2-R-NC-RNA126 non-complementary sequence; **c** SNP (CoV-2-S-DNA72-SNP) and complementary (CoV-2-S-DNA72) sequence and **d** samples of SARS-CoV-2 infected patients P1 (19 Cts), P2 (22 Cts), P3 (25 Cts), P4 (32Cts) and non-infected patient used as control (Control). Data are presented as mean ± SD (*n* = 3) in terms of relative signal increase vs the control. Statistical analysis was performed using either ANOVA (Tukey’s test) or Student’s *T* test. Significant codes: ANOVA 0 “***” 0.001 “**” 0.01 “*” 0.05 “.” 0.1 “” 1 “.” and *T* test *p* value < 0.05 (sig)

Based on the good results obtained with the prototype, we tested our biosensor to detect a DNA sequence of 74 nts. Figure 4a shows the biosensor response, presented as % of the signal increase, to the SARS-CoV-2 samples compared to a non-complementary sequence (GFP- NC-DNA72). As one can observe, the proposed biosensor can detect the target sequence, with a significant increment of the signal (more than ten times) compared to the response to the non-complementary sequence.

#### Detection of RNA SARS-CoV-2 sequences

The promising results obtained above aimed us to assay the detection of an RNA sequence (100 nts) with the proposed electrochemical biosensor and evaluate the possibility of detecting long RNA sequences, which are more similar to the clinical patient samples. Figure 4b shows the signal increase after the hybridization with the target analyte: a complementary sequence (CoV-2-R-RNA100) or a non-complementary sequence (CoV-2- R- NC-RNA126). As can be seen, the difference in % signal increase to complementary sequences is much higher than the observed for the non-complementary sequence, which demonstrates, without doubt, the ability of the biosensor to detect a long RNA sequence (100 nts). This result paves the way to the application to the direct detection of actual samples, which usually contain thousands of nucleotides.

#### Single nucleotide polymorphism detection

Due to the importance of detecting mutations in SARS-CoV-2 genome (to detect more infective SARS-CoV-2 variants such as Alpha, Beta, Gamma, Delta, and Omicron), we wanted to figure out if the biosensor was able to detect a gene mutation based on a Single Nucleotide Polymorphism (SNPs). For this study, we chose a fragment of the coding sequence of the spike protein of SARS-CoV-2 not totally complementary to the probe since it that has a SNP, CoV-2-S-DNA72-SNP sequence (see Table 1SI).

As can be observed in Fig. 4c, the biosensor response to the SNP containing sequence (CoV-2-S-DNA72-SNP), obtained from cells infected with the Wuhan strain (D614)[24], is much lower than to the totally complementary sequence (CoV-2-S-DNA72), obtained from cells infected with the predominant strain in Europe during 2020 (G614), with a difference that allows to affirm that the biosensor is able to detect a SNP without doubt and therefore the mutant forms based on a SNP. This result confirms the high selectivity of the proposed biosensor again.

#### SARS-CoV-2 direct detection in COVID-19 patient samples

Dependable, specific, and rapid diagnostic methods for severe acute respiratory syndrome coronavirus SARS-CoV-2 detection are needed to promote public health interventions for coronavirus disease 2019 (COVID-19). Hence, we took a step forward and applied our methodology to detect SARS-CoV-2 directly in nasopharyngeal swab samples from COVID-19 patients with different viral load, without any amplification process. Samples were previously treated and analyzed by RT-qPCR at Hospital Ramon y Cajal and it served to validate the biosensor. In particular, 4 different nasopharyngeal samples of infected patients, P1 (19 Cts), P2 (22 Cts), P3 (25 Cts), and P4 (32 Cts) were analyzed. A sample from a non-infected patient was used as control (Control).

The direct analysis of the sample from P1 (high viral load) gave the highest biosensor response (around 15% signal increase), whereas samples P2, P3, P4 with a lower viral load gave smaller responses (Fig. 4d). A negligible increase was obtained for the non-infected patient sample (Control). Statistical analysis shows that samples P1, P2, and P3 are significantly different that the control. Thus, we can conclude that the method can clearly discriminate between non-infected and infected patient samples as well as patient samples with different viral load. However, in the case of very low viral load (P4 with 32 Cts), statistical analysis shows non- significant differences with the control.

Note that, these results correlate well with those obtained by RT-qPCR (see Fig. 10SI), highlighting the potential of the approach. In this sense, we must note that the biosensor, like the gold standard technique RT-qPCR, responds well even in patients with different viral load, but unlike RT-qPCR without the need of any amplification process.

The developed biosensor compared well, concerning selectivity, sensitivity, and reproducibility with other electrochemical biosensors previously described to detect targets related to SARS-CoV-2 (see Table 1). The detection limit is comparable or even better than those previously reported in the literature. As can be observed in Table 1, despite some of these authors present biosensors applied to clinical samples with detection limits similar to the proposed in this work [10–12], are in general strategies more complicated and laborious. Our methodology is simple, low cost, and rapid. In addition, our biosensor allows RNA detection of long sequences as well as mutation (SNP) detection. Moreover, the results obtained demonstrate the proposed biosensor can be used to detect SARS-CoV-2 directly in samples from COVID-19 patients without any previous amplification process. This result has great potential in practical applications as an alternative to the classical methods for detecting SARS-CoV-2.

## Conclusions

We developed a gold nanotriangle AuNT-nanostructured based electrochemical biosensor for the sensitive assay of DNA and RNA sequences from genes related to SARS-CoV-2 (RdRp and Spike). The AuNTs enhance the sensitivity and allow the direct immobilization of the corresponding dithio DNA capture probe, which recognized DNA and RNA related to the virus. The hybridization detection is achieved by using Azure A as electrochemical indicator. The system can detect with high sensitivity and selectivity sequences of 25, 74, and 100 nts. Interestingly, it can be used to discriminate SNP present in the Spike gene. Finally, the system was employed to detect samples from patients. The results demonstrate that the developed methodology can clearly discriminate between non-infected and infected patient samples as well as patient samples with different viral load without the need for any amplification method. It is worth to note the broad applicability of the biosensor proposed since it can be applied, changing only the probe, to detect any other DNA/RNA sequence related to other virus or pathogens.

## Supplementary Information

Below is the link to the electronic supplementary material.

Supplementary file1 (DOCX 10390 KB)
