# Supplementary material for: Amplification-free detection of SARS-CoV-2 using gold nanotriangles functionalized with oligonucleotides
Source: Mikrochim Acta. 2022 Apr 1;189(4):171. doi: 10.1007/s00604-022-05272-y (PMC8974806; doi:10.1007/s00604-022-05272-y)
Supplement: Supplementary file 1 — Supplementary file1 (DOCX 10390 KB) [file 604_2022_5272_MOESM1_ESM.docx]

**Electronic Supplementary Material**

**Amplification-Free Detection of SARS-CoV-2 using Gold Nanotriangles Functionalized with Oligonucleotides**

Rafael del Caño ^a,c^, Tania García-Mendiola ^a,b *^, Daniel García-Nieto ^d^, Raquel Álvaro ^d^, Mónica Luna ^d^, Hernán Alarcón Iniesta ^e^, Rocío Coloma ^e^, Ciro Rodríguez Diaz ^e^, Paula Milán-Rois ^e^, Milagros Castellanos ^e^, Melanie Abreu  ^f^, Rafael Cantón  ^f,g^, Juan Carlos Galán  ^f,h^, Teresa Pineda ^c^, Félix Pariente ^a^, Rodolfo Miranda ^e^, Álvaro Somoza ^e^ and Encarnación Lorenzo ^a,b^^,e*^

*^a^ Departamento de Química Analítica. Universidad Autónoma de Madrid. 28049, Madrid (Spain).*

*^b^ Institute for Advanced Research in Chemical Sciences (IAdChem), Universidad Autónoma de Madrid, Ciudad Universitaria de Cantoblanco, 28049, Madrid (Spain).*

*^c^ Departamento de Química Física y Termodinámica Aplicada e Instituto Universitario de Nanoquímica. Universidad de Córdoba. 14014 Córdoba (Spain).*

*^d^ Instituto de Micro y Nanotecnología IMN-CNM, CSIC (CEI UAM+CSIC), Isaac Newton 8, Tres Cantos, 28760, Madrid (Spain).*

*^e^ IMDEA-Nanociencia, Ciudad Universitaria de Cantoblanco, 28049, Madrid (Spain).*

*^f^ Servicio de Microbiología, Hospital Universitario Ramón y Cajal and Instituto Ramón y Cajal de Investigación Sanitaria (IRYCIS), 28034, Madrid (Spain).*

*^g^ Red Española de Investigación en Patología Infecciosa (REIPI), Instituto de Salud Carlos III. Madrid (Spain).*

*^h^ Centro de Investigación Biomédica en Red (CIBER) en Epidemiología y Salud Pública, Instituto de Salud Carlos III. Madrid (Spain).*

*Corresponding author: tania.garcia@uam.es,[encarnacion.lorenzo@uam.es](mailto:encarnacion.lorenzo@uam.es)

**Experimental details**

**Table 1SI**. DNA/RNA sequences used in this work.

|  | **SARS-CoV-2 DNA/RNA sequences** | | **Named** |
| --- | --- | --- | --- |
| Probe R thiol | | 5´-GCATCTCCTGATGAGGTTCCACCTG- thiol | Probe-R-DNA-thiol |
|  | | 5´-TAMRA-GCATCTCCTGATGAGGTTCCACCTG- thiol | Probe-R-TAMRA-thiol |
| Probe R dithiol | | 5´-GCATCTCCTGATGAGGTTCCACCTG- dithiolane | Probe-R-DNA-dithio |
| Probe S dithiol | | 5´-GTGCAGTTAACACCCTGATAAAGAA- dithiolane | Probe-S-DNA-dithio |
| Targets | | 5´-CAGGTGGAACCTCATCAGGAGATGC | CoV-2-R-DNA25 |
|  |  | 5´-GTGTGGCGGTTCACTATATGTTAAACCAGGTGGAAC  CTCATCAGGAGATGCCACAACTGCTTATGCTAATAGTG | CoV-2-R-DNA74 |
|  | | 5´-GGGGUGAAAUGGUCAUGUGUGGCGGUUCACUAUA  UGUUAAACCAGGUGGAACCUCAUCAGGAGAUGCCAC  AACUGCUUAUGCUAAUAGUGUUUUUAACAUUUG | CoV-2-R-RNA100 |
|  | | 5´-CAAATACTTCTAACCAGGTTGCTGTTCTTTATCAGG  GTGTTAACTGCACAGAAGTCCCTGTTGCTATTCATG | CoV-2-S-DNA72 |
|  | | 5´-CAAATACTTCTAACCAGGTTGCTGTTCTTTATCAGG  **A**TGTTAACTGCACAGAAGTCCCTGTTGCTATTCATG | CoV-2-S-DNA72-SNP |
| Non Complementary | | 5´-ACACTAGCCATCCTTACTGCGCTTCG | CoV-2-E-NC-DNA25 |
|  | | 5´-CACAACGTCTATATCATGGCCGACAAGCAGAAGAA  CGGCATCAAGGTGAACTTCAAGATCCGCCACAACATC | GFP- NC-DNA72 |
|  | | 5´-GAGACAUUGGCCGUGACAGCUUGACAAAUGUUAA  AAACACUAUUAGCAUAAGCAGUUGUGGCAUCUCCUGAUGAGGUUCCACCUGGUUUAACAUAUAGUGAACCGCCACACAUGACCAUUUCAC | CoV-2- R- NC-RNA126 |

***DNA/RNA samples.***

Short DNA and RNA sequences were synthesized in a H-6/H-8 DNA/RNA Synthesizer (K&A) and purified using LCG Biosearch Micropure purification columns. The capture probes R and S used were modified at the 3’-end with a standard thiol modification (thiol) or a dithiolane derivative (dithio) [1]. The synthesis was done using the DMT-ON method, to ease their purification. The oligonucleotides were cleavaged from the solid support by the incubation with ammonium solution (30%) for 18 hours. The ammonium was eliminated by vacuum, and the solutions were introduced in the purification columns. All the purification process was performed with the P-8 purifier according to the recommendations of the manufacturer. Finally, the oligonucleotides were concentrated and quantified.

Longer single-stranded DNA molecules (72-74bp) were obtained by RT-PCR. Briefly, total RNA extracted from infected cell cultures were amplified using the SuperScript™ III One-Step RT-PCR System with Platinum™ Taq DNA Polymerase (Invitrogen). Several reactions of 50 µl each were prepared according to the manufacturer’s instructions with the following modifications: 20 ng of total RNA as template and 1 µM forward primer (containing the first 6 nucleotides phosphorothioate modifications), 1.2 µM reverse primer (Table 1SI), and 2 mM MgSO_4_). The cycling parameters were adjusted to the specific amplified region and summarized in Table 1SI. Amplified RT-PCR products for the same target region were pooled and loaded in a 3% agarose gel to check the success of the reaction. Finally, the dsDNA products were treated with 0.4 U T7 exonuclease (New England Biolabs) per µl of RT-PCR reaction during 45 min at 37 ºC followed by 10 min at 80ºC, to digest the unprotected (non-phosphorothioated) strand. Finally, ssDNA was purified using the Monarch PCR & Cleanup Kit (New England Biolabs). The purified ssDNA product was resuspended in PCR-grade water and used as target in detection experiments.

As negative control, a region of the GFP gene was amplified and purified following the same procedures as longer ssDNA molecules described above but with some modifications. RT amplification step was avoided, and the reaction mix contained 1 µM of each forward and reverse primer and 20 ng of plasmid DNA pEGFP-C1 (Takara).

The ≥ 100bp RNA fragments were synthesised using the MEGAscript T7 Transcription Kit (Invitrogen). First, the target sequence was PCR amplified from plasmids pUC57_F4 (kindly provided by Isabel Sola and Sonia Zuñiga), containing the sequence of a region of the SARS CoV 2 RdRp and pSL_WH_SARS2_S, containing the sequence of SARS CoV 2 S protein. Next, amplification was performed with oligos carrying the T7 RNA polymerase DNA-dependent promoter (**Table 1SI**). Once the target regions were amplified, the DNA was used as a template for T7 polymerase in a standard in vitro transcription reaction 16h at 37 ^o^C. The RNA product was purified according to the kit instructions, analysed by polyacrylamide-urea gel electrophoresis and quantified in a NANOdrop apparatus.

**R 74 bp**

| Temperature ºC | Time | Cycles |
| --- | --- | --- |
| 55 | 15 min | 1 |
| 94 | 2 min | 1 |
| 94 | 15 s | 35 |
| 56,5 | 30 s |  |
| 68 | 10 s |  |
| 68 | 5 min | 1 |
| 4 | ∞ | 1 |

**S 72 bp**

| Temperature ºC | Time | Cycles |
| --- | --- | --- |
| 55 | 15 min | 1 |
| 94 | 2 min | 1 |
| 94 | 15 s | 35 |
| 54 | 30 s |  |
| 68 | 10 s |  |
| 68 | 5 min | 1 |
| 4 | ∞ | 1 |

**GFP 72 bp**

| Temperature ºC | Time | Cycles |
| --- | --- | --- |
| 94 | 2 min | 1 |
| 94 | 15 s | 35 |
| 54 | 30 s |  |
| 68 | 10 s |  |
| 68 | 5 min | 1 |
| 4 | ∞ | 1 |

**R 100 bp**

| **Temperature ºC** | **Time** | **Cycles** |
| --- | --- | --- |
| 94 | 2 min | 1 |
| 94 | 15 s | 30 |
| 58 | 30 s |  |
| 72 | 10 s |  |
| 72 | 4 min | 1 |
| 4 | ∞ | 1 |

**Table 2SI**. Primers used in the amplification process.

| Entry | Name | Sequence 5'-->3' | Amplified sequence and length |
| --- | --- | --- | --- |
| 1 | Fwd-pt RdRP | G*T*G*T*G*G*CGGTTCACTATATGTT | R 74 nts |
| 2 | Rv-OH RdRP | GCACTATTAGCATAAGCAGTTGTG | R 74 nts |
| 3 | Fwd-pt S | C*A*A*A*T*A*CTTCTAACCAGGTTGC | S 72 nts |
|  | Rv-OH S | CATGAATAGCAACAGGGACTTC | S 72 nts |
| 4 | Fwd-pt GFP | C*A*C*A*A*C*GTCTATATCATGGC | GFP 72 nts |
| 5 | Rv-OH GFP | GATGTTGTGGCGGATCTTGA | GFP 72 nts |
| 6 | Fwd-RdRP-100 | TAATACGACTCACTATAGGGGTGAAATGGTCATGTGTGGCGG | R 100 nts |
| 7 | Rv-RdRP-100 | CAAATGTTAAAAACACTATTAGCATA | R 100 nts |
| 8 | Fwd-RdRP-126 | AATTCTAATACGACTCACTATAGGGAGACATTGGCCGTGACAGCTTGACAAATG | R 126 nts |
| 9 | Rv-RdRP-126 | GTGAAATGGTCATGTGTGGCGG | R 126 nts |

* denotes phosphorothioate

***Apparatus.***

For transmission electron microscopy (TEM), Lacey carbon support film copper grids (400 mesh, Electron Microscopy Sciences) were used. Images were recorded with a JEOL JEM 2100 electron microscope.

UV-Vis spectra were recorded using a Jasco V-670 UV-vis-NIR spectrophotometer using a quartz cell.

Raman spectroscopy measurements were recorded on a Bruker Senterra confocal Raman microscope (Bruker Optic, Ettlingen, Germany, resolution 3–5 cm^-1^) by using the following parameters: objective NA 0.75, 50X; laser excitation: 532 nm, 2 mW. Each spectrum results from the average of 3 measurements carried out in different regions distributed all over the sample. Optical microscope images were carried out using an Olympus microscope with a 50x objective lens.

SEM images of the electrodes were characterized working on a low voltage (2.5 kV) and current (10 pA) mode to avoid any damage of the sample by using a FESEM Auriga, Carl Zeiss equipped with an energy-dispersive X-ray spectrometer.

Atomic Force Microscopy (AFM) images were acquired with a Nanotec Electrónica AFM system, in non-contact mode, using silicon cantilevers (PPP-FM Nanosensors, 2.8 N/m nominal spring constant and 75 kHz resonant frequency). WSxM software [2] was used for data acquisition and image processing.

Fluorescence microscopy was performed with an incident light microscope Axioskop 2 MAT (ZEISS), implemented with a mercury short arc lamp HBO 50 W/AC L1 (OSRAM).

AuNTs SEM images were taken at a working voltage of 1 kV and a current of 25 pA in a Scanning Electron Microscope VERIOS 460 from FEI.

Dynamic light scattering (DLS) and Zeta-potential analysis were performed using a Malvern Zetasizer Nano, ZSP with 633 nm He-Ne laser, equipped with an MPT-2 Autotitrator. The measured data are the average of at least 20 runs. The average hydrodynamic diameter and mean zeta potential of each sample were computed using the software provided by the manufacturer.


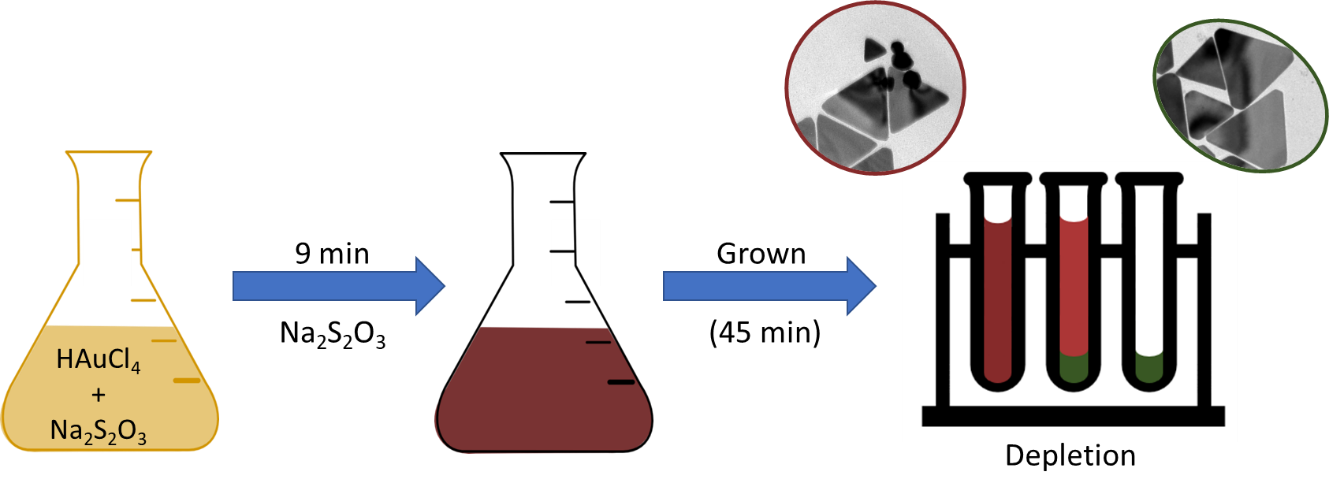


**Scheme1 SI**. Scheme of the AuNTs synthesis.

**Fig. 1SI**. SEM images of AuNTs deposited on a highly oriented pyrolytic graphite (HOPG) substrate at different magnifications (a, b,c and d). Zeta potential (e) and size (f) of AuNTs at different pH values.

**Fig. 2SI**. SEM (a) and EDX images (b) of a magnification of a CSPE. Scale bar corresponds to 600 µm. c) EDX spectrum of a AuNTs/CSPE.

**Fig. 3SI**. Optical microscope white light image (a) and fluorescence image (b) of AuNTs/ CSPE.


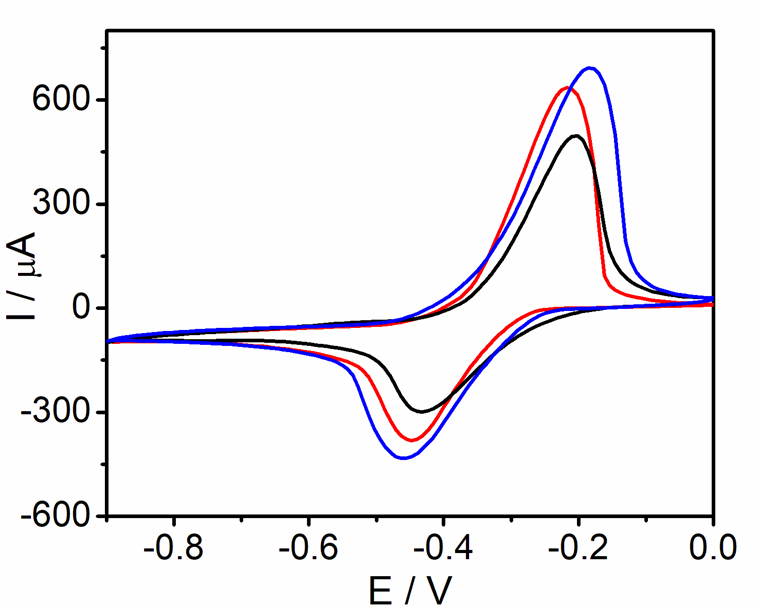


**Fig. 4SI**. Cyclic voltammograms from -0.9V to 0V of 10 mM AA in 0.1M PB pH 7.0 at a CSPE (black line), AuNTs/CSPE (red line) and Probe R-DNA-thiol/AuNTs/CSPE (blue line). Scan rate: 100 mVs^-1^.


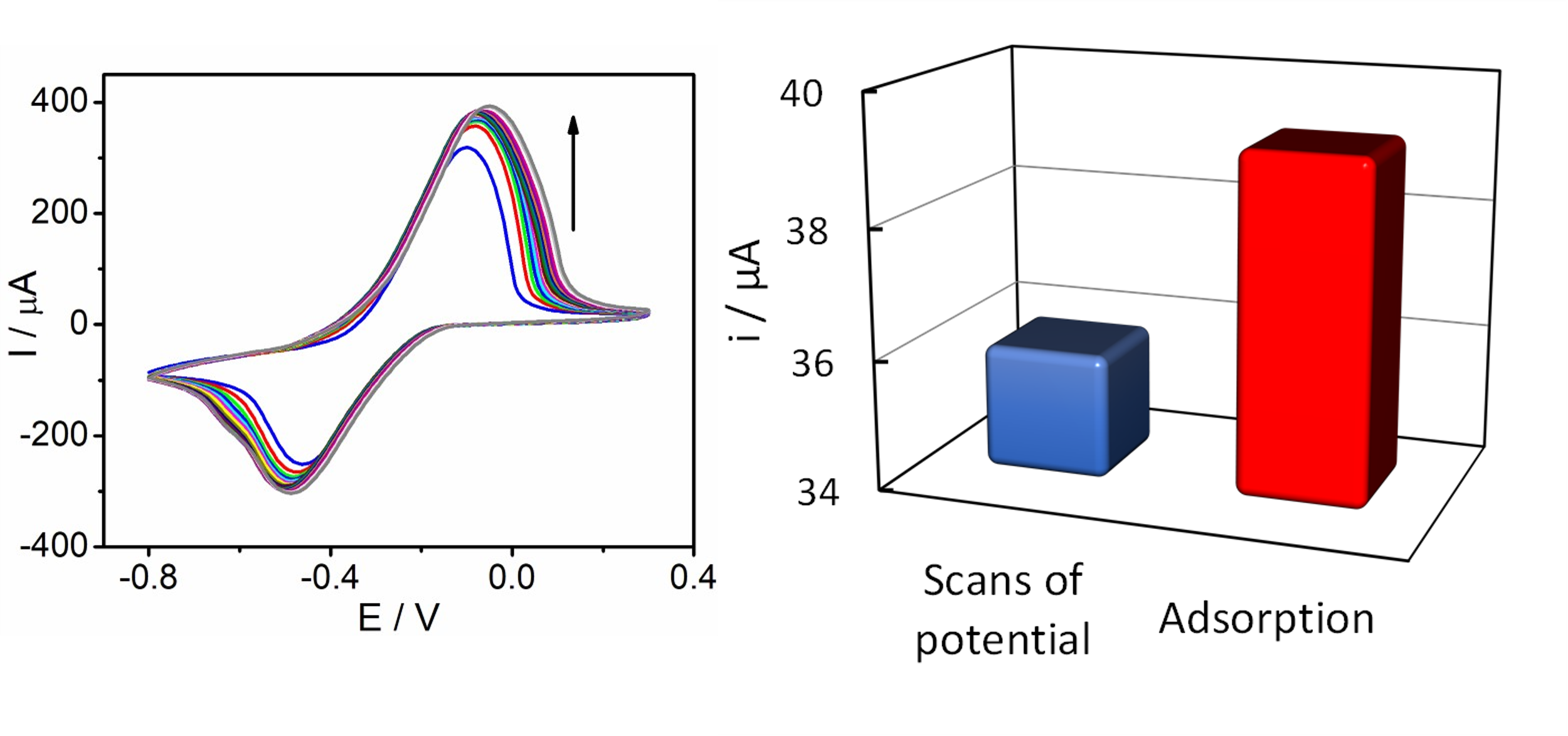


**Fig. 5SI**. (a) CVs at the dsDNA-AuNT-CSPE in a 10 mM AA in 0.1M PB pH 7.0. (b) Bar diagram of the oxidation current of AA accumulated on the dsDNA layer by cycling potential (blue bar) or by adsorption (red bar).

**
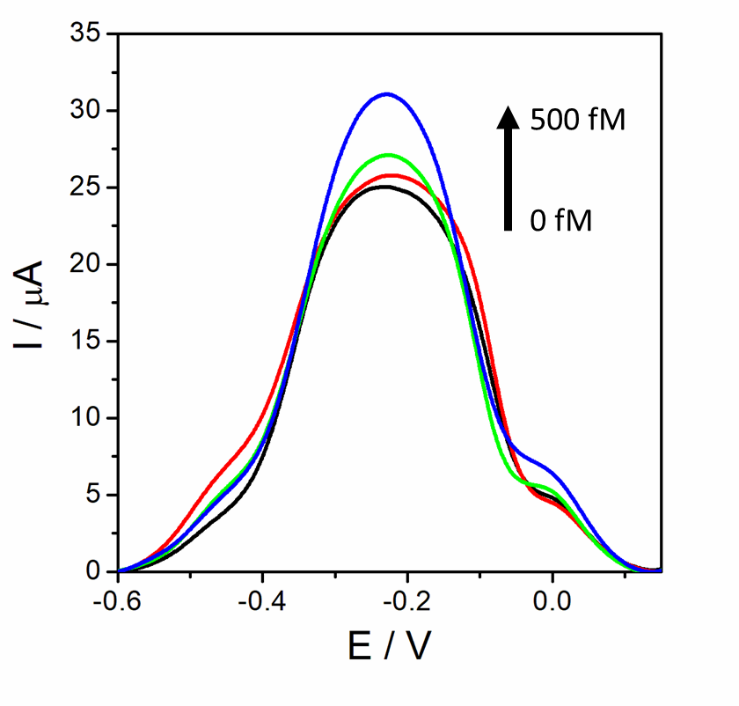
**

**Fig. 6SI.** DPVs in PB 0.1 M pH 7.0 of the AA accumulated on a Probe-R-DNA-dithio/AuNT/CSPE) after hybridization with different concentrations ( 0 to 500 fM, calibration plot) of complementary CoV-2-R-DNA25 sequence. Scan rate: 10 mV s^-1^.

**Fig. 7SI**.Bar diagrams of the biosensor response after hybridization with: 50.0 fM of complementary SARS-CoV-2_C_ (red), a mixture of 50.0 fM of SARS-CoV-2_C_ and 50.0fM of Influenza A sequences (green) and a mixture of 50.0 fM of SARS-CoV-2_C_ and 50.0fM of SARS-CoV-1 sequences (blue).


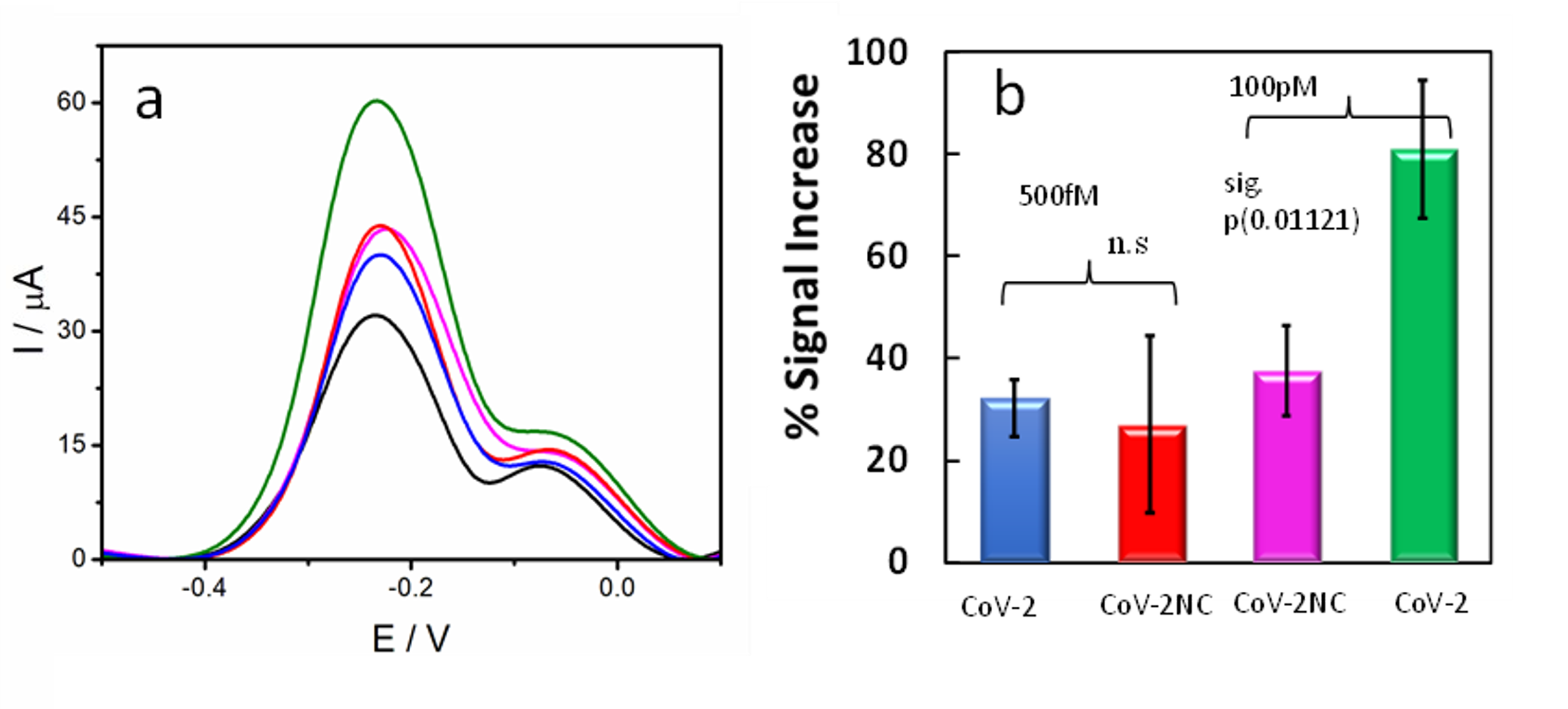


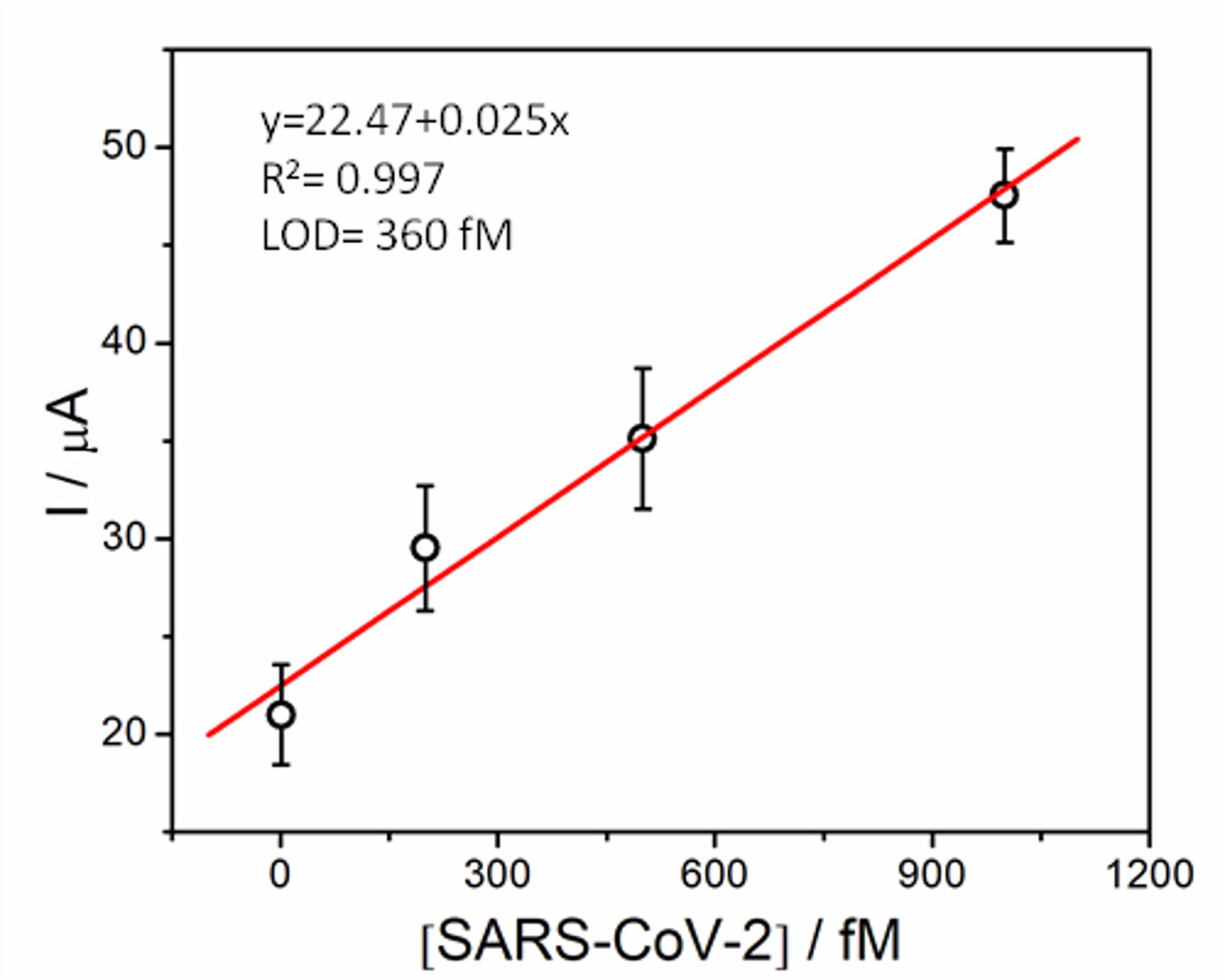
**Fig. 8SI**. DPVs (a) and bar diagrams (b) of the peak current of AA accumulated on a Probe-R-DNA-dithio/AuNPs/CSPE after hybridization with 500 fM of complementary CoV-2-R-DNA25 (blue curve) and a non-complementary CoV-2-E-NC-DNA25 (red curve) sequences or 100 pM of CoV-2-R-DNA25 (green curve) and a non-complementary CoV-2-E-NC-DNA25 (purple curve) in PB 0.1 M pH 7.0. Scan rate: 10 mV s^-1^. Data are presented as mean ± SD (n=3) in terms of percentage of signal increase. Statistical analysis was performed using a Student T-test (independent samples) with 95% confidence interval and α = 0.05. Significative differences for p-value < 0.05 (sig).

**Fig. 9SI**. Calibration plot of the biosensor nanostructured with spherical gold nanoparticles (AuNPs) to different concentrations of SARS-CoV-2 sequence (1.00 to 1000 fM).

**Fig. 10SI**. Correlation between biosensor response (y-axis) versus Cts performed by RT-qPCR (x-axis) for samples of SARS-CoV-2 infected patients P1 (19 Cts), P2 (22 Cts), P3 (25 Cts) and P4 (32Cts).

**References**

1. Latorre A, Posch C, Garcimartan Y, et al (2014) Single-point mutation detection in RNA extracts using gold nanoparticles modified with hydrophobic molecular beacon-like structures. Chemical Communications 50:3018–3020. https://doi.org/10.1039/c3cc47862a

2. Horcas I, Fernández R, Gómez-Rodríguez JM, et al (2007) WSXM: A software for scanning probe microscopy and a tool for nanotechnology. Review of Scientific Instruments 78:13705. https://doi.org/10.1063/1.2432410
